# Current Practice in FFP Preparation and Use in Greece: A National Survey

**DOI:** 10.4274/tjh.galenos.2020.2020.0241

**Published:** 2021-02-25

**Authors:** Aspasia Argyrou, Serena Valsami, Abraham Pouliakis, Maria Gavalaki, Antonis Aggelidis, Vasiliki Voulgaridou, Vasiliki Pliatsika, Theofanis Adraktas, Andreas Papachronis, Chrysoula Alepi, Vasiliki Giannopoulou, Panagiotis Siourounis, Sofia Tsagia, Georges Martinis, Eftihia Kontekaki, Eleftheria Zervou, Spiridon Koliofotis, Elias Kyriakou, Athina Mougiou, Lempousi Dimitra, Afrodite Chairopoulou, Aggeliki Tsakania, Maria Baka, Ioanna Apostolidou, Dimitra Moschandreou, Anastasia Livada, Marianna Politou, Fragoula Roussinou, Christina Pappa, Vasiliki Koika, Niki Vgontza, Anthippi Gafou, Ioanna Dendrinou, Fotini Sakellaridi, Lampothea Labrianou, Zafeiria Alexandropoulou, Vasiliki Sochali, Kostas Malekas, Areti Skordilaki, Georgia Kakava, Konstantinos Lebesopoulos, Konstantinos Stamoulis, Elisavet Grouzi

**Affiliations:** # These authors contributed equally to this work.; 1 Agioi Anargyroi Hospital, Department of Blood Transfusion, Athens, Greece; 2 National and Kapodistrian University of Athens, Aretaieion University Hospital, Medical School, Hematology Laboratory-Blood Bank Department, Athens, Greece; 3 National and Kapodistrian University of Athens, Attikon University Hospital, Second Department of Pathology, Athens, Greece; 4 National and Kapodistrian University of Athens, Attikon University Hospital, Second Department of Pathology, Athens, Greece; 5 Konstantopouleio-Neas Ionias General Hospital, Department of Blood Transfusion, Athens, Greece; 6 AHEPA, University Hospital, Department of Blood Transfusion, Thessaloniki, Greece; 7 General Hospital Tzaneio, Department of Blood Transfusion, Piraeus, Greece; 8 Agios Panteleimon General Hospital of Nikaia, Department of Blood Transfusion, Athens, Greece; 9 University Hospital, Department of Blood Transfusion, Alexandroupolis, Greece; 10 University Hospital, Department of Blood Transfusion, Ioannina, Greece; 11 Attikon University Hospital, Laboratory of Hematology and Blood Bank Unit, Athens, Greece; 12 University Hospital, Blood Transfusion Center, Patras, Greece; 13 Sismanogleio General Hospital, Department of Blood Transfusion, Athens, Greece; 14 Thriasio General Hospital, Department of Blood Transfusion, Athens, Greece; 15 Saint Savvas Oncology Hospital, Department of Blood Transfusion and Clinical Hemostasis, Athens, Greece; 16 General Hospital, Department of Blood Transfusion, Korinthos, Greece; 17 General Hospital, Department of Blood Transfusion, Rhodes, Greece; 18 Asklipieio Voulas General Hospital, Department of Blood Transfusion, Athens, Greece; 19 General Hospital, Department of Blood Transfusion, Giannitsa, Greece; 20 General Hospital, Department of Blood Transfusion, Livadia, Greece; 21 General Hospital, Department of Blood Transfusion, Chania, Greece; 22 Pammakaristos General Hospital, Department of Blood Transfusion, Athens, Greece; 23 Amalia Fleming General Hospital, Department of Blood Transfusion, Athens, Greece; 24 Hellenic National Blood Transfusion Center, Athens, Greece

**Keywords:** Transfusion medicine, Acquired coagulopathies, Replacement therapies, Blood coagulation

## Abstract

**Objective:**

Fresh frozen plasma (FFP) transfusion is widely used in modern clinical settings. Practices regarding its use vary due to lack of guidelines from randomized trials. The aim of this study was to assess both the current practices regarding FFP production, use, and wastage and the implementation of quality control (QC), female donor plasma production policies, and use of pharmaceutical hemostatic agents in Greece.

**Materials and Methods:**

The study was conducted during February-April 2018. For the first part of the study, data including FFP transfusion indication, hospital department, diagnosis, FFP units/transfusion episode, ABO compatibility, blood donor’s sex, and reasons for discarding were collected. For the second part, questionnaire data were analyzed.

**Results:**

According to data from 20 Greek hospitals, 12655 FFP units were transfused to 2700 patients during 5069 transfusion episodes in the studied period of time. Most patients were hospitalized in internal medicine, general surgery, and intensive care unit departments. Each patient received on average 4.69 units (2.5 units/episode). Transfusion requests were in accordance with international guidelines in 63.44% of cases and 99.04% of the units were given to ABO-identical patients. Main reasons for discarding included failure to meet quality requirements (30.06%), female donors (22.17%), and other causes (27.26%). Among 96.9% of all transfusion services across the country, 28.26% perform QC according to the directions of the European Directorate for the Quality of Medicines & Health Care and 68.83% discard plasma from female donors. Pharmaceutic hemostatic agents are used in 37.23% of the hospitals.

**Conclusion:**

This is the first national survey regarding FFP production and transfusion in Greece. Staff of internal medicine, general surgery, and ICU departments, where most FFP-transfused patients are hospitalized, should be regularly involved in training on contemporary transfusion guidelines. Upcoming centralization of FFP production and inventory management could help in homogenizing practices regarding FFP use and improve product quality. Strengthening the use of pharmaceutic hemostatic agents could improve patients’ management.

## Abstract (tr)

**Amaç:**

Taze donmuş plazma (TDP) transfüzyonu modern klinik ortamlarda yaygın olarak kullanılmaktadır. Kullanım ile ilgili uygulamalar, randomize çalışmalardaki kılavuzların eksikliğinden dolayı değişiklik gösterir. Bu çalışmanın amacı, Yunanistan’da TDP üretimi, kullanımı ve israfına ilişkin hem mevcut uygulamaları hem de kalite kontrolünün uygulanmasını, kadın donör plazma üretim politikalarını ve farmasötik hemostatik ajanların kullanımını değerlendirmekti.

**Gereç ve Yöntemler:**

Çalışma, Şubat-Nisan 2018’de gerçekleştirildi. Çalışmanın ilk bölümü için TDP transfüzyon endikasyonu, hastane bölümü, tanı, TDP üniteleri/transfüzyon sayısı, ABO uyumluluğu, kan vericinin cinsiyeti ve dışlama nedenleri gibi veriler toplandı. İkinci bölüm için anket verileri analiz edildi.

**Bulgular:**

Yirmi Yunan Hastanesi’nden alınan verilere göre, incelenen zaman diliminde 5069 transfüzyon sırasında 12655 TDP ünitesi 2700 hastaya nakledildi. Hastaların çoğu dahiliye, genel cerrahi ve yoğun bakım ünitelerinde yatırıldı. Her hasta ortalama 4,69 ünite (2,5 ünite/transfüzyon) aldı. Olguların %63,44’ünde transfüzyon talepleri uluslararası yönergelere uygun olup, ünitelerin %99,04’ü ABO özdeş hastalara verilmiştir. Dışlamanın temel nedenleri arasında kalite gereksinimlerini karşılamama (%30,06), kadın donörler (%22,17) ve diğer nedenler (%27,26) yer aldı. Ülke çapındaki tüm transfüzyon hizmetlerinin %96,9’u arasında, %28,26’sı Avrupa İlaç ve Sağlık Hizmetleri Kalitesi Direktörlüğü’nün talimatlarına göre kalite kontrol gerçekleştirmekte ve %68,83’ü kadın donörlerden plazmayı dışlamaktadır. Hastanelerin %37,23’ünde farmasötik hemostatik ajanlar kullanılmaktadır.

**Sonuç:**

Bu çalışma, Yunanistan’da TDP üretimi ve transfüzyonu ile ilgili ilk ulusal ankettir. TDP ile transfüzyon yapılan hastaların çoğunun hastaneye kaldırıldığı dahiliye, genel cerrahi ve yoğun bakım ünitesi departmanlarının personeli, çağdaş transfüzyon kılavuzları konusunda düzenli olarak eğitime dahil edilmelidir. TDP üretiminin ve envanter yönetiminin yakında merkezileştirilecek olması, TDP kullanımına ilişkin uygulamaların homojenleştirilmesine ve ürün kalitesinin iyileştirilmesine yardımcı olabilir. Farmasötik hemostatik ajanların kullanımının güçlendirilmesi, hastaların yönetimini iyileştirebilir.

## Introduction

Fresh frozen plasma (FFP) is used for patients with abnormal coagulation test results due to consumption or decreased production of coagulation factors, who either bleed (therapeutic use) or undergo invasive procedures or surgery (prophylactic use). FFP transfusion is also indicated for immediate anti-vitamin K-reversal, for patients with thrombotic thrombocytopenic purpura (TTP), and for congenital coagulation factor deficiencies when alternative therapies are not available [1].

These indications are described in currently used guidelines in many countries, including Greece [2,3], but they are not supported by evidence from high-quality randomized trials [2,3]. This fact together with the underestimation of the adverse effects of FFP use often result in a lack of strict adherence to FFP usage guidelines. Notably, the percentage of inappropriate FFP requests varies between 10% and 73% according to published data [4].

This study was conducted in order to assess and evaluate current trends in FFP production and use in Greece (request etiology, dosage/transfusion episode, cases of inappropriate use, discards). We also evaluated the implementation of FFP quality control (QC), female donor plasma production policies, and the use of pharmaceutical hemostatic agents. Having established the current status in our country, we could develop effective educational strategies to achieve higher compliance with the international guidelines.

## Materials and Methods

This study was conducted by the Working Committee of Transfusion and Apheresis of the Hellenic Society of Hematology during a 3-month period (February-April 2018) and all 97 blood transfusion services (BTSs) in Greece were invited to participate.

It consists of two parts; the first part focuses on FFP origin, use, and disposal. An electronic data collection form (Excel 2016, Microsoft Corp., Redmond, WA, USA) was used. Recorded data included i) FFP data (transfusion date, etiology, number of transfusion episodes, FFP units’ ABO group, origin - male/female donor, produced in-house/imported), ii) patients’ data [age, ABO group, clinical department, diagnosis, pre-transfusion international normalized ratio (INR)], and iii) data about discarded FFP units (ABO group, etiology, date of discarding). All participants were provided with a coded list of FFP transfusion indications (Table 1). The transfusion of several plasma units on the same day is defined as a “single transfusion episode.” Data regarding national plasma units’ supplies and usage were provided by the Hellenic National Blood Transfusion Center.

The second part of the study comprises three questions. Participating BTSs answered them by filling out the electronic data form or responded by means of a phone interview by a member of the Working Committee. The aim here was to assess i) whether QC of the produced plasma units was performed according to the requirements of the European Directorate for the Quality of Medicines & Health Care (EDQM) [5], ii) local policies regarding the production of plasma from female donors, and iii) the use of pharmaceutical hemostatic agents [prothrombin complex concentrate (PCC), fibrinogen concentrate, recombinant activated factor VII (rVIIa)].

### Statistical Analysis

Electronic spreadsheet data forms (Excel 2016, Microsoft Corp., Redmond, WA, USA) and SAS software version 9.4 for Windows (SAS Institute Inc., Cary, NC, USA) were used for the statistical analysis. For all tests the significance level was set to p<0.05 and the confidence interval (CI) to 95%. The results are reported as mean value and standard deviation (SD) for the arithmetic parameters and as percentages for the categorical.

## Results

Data were collected from 20 of the 97 BTSs (20.62%) invited to join this study. Twelve are located in Athens and the remaining eight are located in other cities (Thessaloniki, Patras, Ioannina, Alexandroupolis, Korinthos, Rhodes, Livadia, Edessa).

A total of 12655 FFP units were transfused to 2700 patients in 5069 transfusion episodes. Given that the number of FFP units annually transfused in Greece is approximately 250000, the number of units transfused over this 3-month period is representative of the corresponding annual consumption (CI 95%, error margin 0.85%). The number of FFP units used by hospitals located in Athens was 7620 (60.2%) and the remaining 5035 (39.8%) units were used by hospitals in the rest of the country. FFP units transfused in every participating hospital, the number of patients who received FFP, the number of transfusion episodes, and the number of units/episode in each hospital are listed in Table 2.

Each patient received an average of 4.7 units (range: 1-280, SD: 12.6). The average number of episodes/patient was 1.88±2.33, while a mean number of 2.5±2.61 units was used in each episode.

The majority of the units were transfused to patients admitted to internal medicine (28.57±0.79%), surgery (16.02±0.64%), ICU (15.79±0.64%), neurological (5.65±0.41%), and hematology (3.99±0.35%) departments, while 9.22±0.51% of the units were transfused in private hospitals (Table 3).

Patients with solid tumors (n=429) received the most FFP units (1780 units, 14.07±0.61%, 4.15 units/patient), followed by patients with sepsis (n=241 patients, 1101 units [8.70±0.49%], 4.57 units/patient), autoimmune diseases (n=28 patients, n=1020 units [8.06±0.48%], 36.43 units/patient), liver disease (n=134 patients, n=817 units [6.46±0.43%], 6.1 units/patient), major gastrointestinal bleeding (n=205 patients, n=668 units [5.28±0.39%], 3.26 units/patient], Guillain-Barre syndrome (n=5 patients, n=430 units [3.40±0.32%], 86 units/patient), and TTP (n=3 patients, n=396 units [3.13±0.31%], 132 units/patient).

Among the predefined indications of Table 1, optimization of coagulation prior to surgery was the main indication of transfusion. FFP transfusion indications, the number of patients and units transfused, the number of transfusion episodes, the number of units/episode, and the episodes/patient are depicted in Table 4.

Coagulation screening tests were available for 3824 out of 5069 (75.44±1.19%) transfusion episodes. Among these, prolonged prothrombin time (INR of ≥1.5) was found in 1514 episodes while the remaining had INR of <1.5. In these two categories of episodes, 3476 (27.47%) and 4970 (39.27%) FFP units were transfused, respectively. No coagulation testing was available for 1255 (24.76±1.19%) episodes in which 4209 (33.36%) of the total units were transfused.

Most patients (n=1598, 59.19±1.86%) were transfused with a total number of ≤2 FFP units, while 1102 patients (40.81±1.86%) received ≥3 units. Most of the patients who received ≤2 units had an unspecified transfusion etiology (n=419), while among those who were given ≥3 units, preoperative FFP transfusion was the most common etiology (n=289) and TTP was the least common one (n=13).

The distribution of FFP units transfused according to ABO blood group is depicted in Table 5.

All participating BTSs in our study, with one exception, collect blood and produce blood components themselves and also import units from other hospitals whenever there is a need to cover increased demands (Table 6). Single-donor plasmapheresis is not routinely performed and plasma is almost exclusively produced from whole blood donations. The great majority of units (n=10537, 83.26±0.65%) were donated by males; 1042 (8.23±0.48%) were donated by females, while for the remaining 1076 (8.5±0.49%), the donor’s sex was not stated.

As for the discarding of the plasma produced, 5538 units were disposed of. Almost half of them (2738 units (49.44±1.32%]) were meant to be destroyed by the time of venipuncture and the rest at a later stage of the procedure (Table 7).

In the second part of the study, 94 out of the total of 97 BTSs (96.9±3.82%) in Greece answered our questions. Regarding the QC of the produced plasma, 26 of the 92 BTSs (28.26±9.06%) to answer this question perform it following the directions of the EDQM [5]. The remaining 66 BTSs (71.74±9.06%) partly follow the above-mentioned directions, mostly due to the unavailability of the necessary medical technology (e.g., analyzers for measuring FVIII levels).

With respect to how plasma from female donors is handled, our study revealed that 60 BTSs (63.83±9.54%) discard plasma from female donors, 27 (28.72±9.00%) produce plasma from female donors with ≤2 pregnancies, 5 (5.32±4.78%) produce plasma with no differentiation between male and female donors, and 2 (2.13±3.42%) do not produce plasma at all.

Concerning the use of hemostatic agents, 35 BTSs (37.23±9.59%) use such medicines, while the remaining 49 (52.13±9.59%) do not. Four BTSs (4.25±4.39%) use other methods to achieve hemostasis (e.g., cryoprecipitate) and 6 (6.38±5.14%) did not answer.

## Discussion

This study represents the first attempt to present the current practice regarding FFP production and use in Greece, depicting the status before the launching of the central inventory management system that the Hellenic National Blood Transfusion Center has already started. Centralization in the blood supply chain is a well-established practice in developed countries worldwide [6,7]. Notably, decentralization in blood collection and the preparation and distribution of blood components leads to a variety of practices affecting many procedures along the blood transfusion chain [8,9].

The distribution of patients grouped by clinical department in Greece (Table 3) does not seem to differ from distributions in other countries (e.g., England), as in other studies most patients were hospitalized in internal medicine, surgical wards, and ICUs [1].

It is worth noting that plasma transfusion trends and policies vary worldwide. In several countries (e.g., UK, Canada, Iran), increased or improper FFP use has been reported. This is in line with our results that revealed a significant level of inappropriate use of this blood component. The lack of strong evidence supporting specific plasma transfusion practices could be a possible explanation [1,10,11,12,13].

In our study, the majority of FFP units (64.85%) were given according to the predefined indications depicted in Table 1, which is in line with the percentages of other studies, ranging from 31.5% to 85% [1,14,15,16,17]. The transfusion of the 15.53% of FFP units that were not compliant with predefined indications (FFP7 category) concerned cases of emergency patients, whose transfusions were mainly based on clinical criteria (e.g., perioperative bleeding, major gastrointestinal bleeding), or patients with hypofibrinogenemia or hypoalbuminemia in hospitals where neither human albumin nor fibrinogen concentrates or cryoprecipitate were available. Similarly, several studies outlined surgery-related conditions (even with mild or no elevated laboratory coagulation tests), hypoalbuminemia, and burns as the most common reasons for FFP use outside of strict indications [14,16,17]. Apart from this inappropriate but justified FFP use, the transfusion of 19.62% of plasma units remained unjustified (FFP8 category), which underlines the need for more consistent documentation of FFP requests by clinicians and the need for reevaluation of FFP requests, possibly by the BTS staff. Implementation of continuous and repeated education regarding FFP transfusion indications and use of electronic clinical decision support systems could help in reducing inappropriate FFP requests [18].

Regarding the FFP doses in our study, our observation of FFP underuse is consistent with what is reported in the international literature [19]. Given that the indicated FFP dose is 10-15 mL/kg, our data show that patients receive less than the recommended volume (2.5 units/episode), with exceptions for patients who undergo plasma exchange and receive the indicated dose.

ABO-identical plasma was given in the vast majority of cases, and only a few ABO-incompatible plasma units (27/12655) were transfused. According to the Hellenic Hemovigilance Center’s annual reports, no case of hemolytic transfusion reaction due to FFP transfusion was recorded from 2012 to 2018 [20,21], which is consistent with data reporting that even when plasma concentrates of “dangerous” universal O blood group donors are given to a non-O patient, the actual incidence of hemolytic transfusion reactions is low [22,23]. Additionally, no significant differences have been found regarding complications and mortality between patients transfused with ABO-compatible and ABO-incompatible plasma [24].

Regarding wastage (Table 7), the most common reasons for discarding units in our study was return from the clinical wards, leakage, and breakage, as previously described. The wastage rate due to expiry date was quite low (0.9%), while in other studies it was higher [12,25,26]. Although wastage underreporting could not be ruled out, this finding may reflect a proper inventory management according to the “first in, first out” (FIFO) policy [27,28].

As for the use of female-origin plasma and plasma-containing components, it has been reported that it has been implicated in the pathogenesis of the majority of transfusion-related acute lung injury (TRALI) fatalities [29]. Several strategies have been implemented in many countries to reduce the risk of TRALI related to female donor deferral, but these are not yet standardized and they differ between individual blood banks [30,31]. Our study also reveals this variety, which is linked to decentralization and reflects not only the lack of concrete national recommendations but also the efforts of some BTSs to ensure an adequate inventory. This practice may be connected to data from the 2018 annual report of the Hellenic National Hemovigilance Center, which shows two FFP-related TRALI cases and one TRALI-related death in 2018 [21]. Nevertheless, we cannot confirm that those cases took place within the 3-month period of our study.

Regarding plasma QC, many countries follow the World Health Organization’s recommendation for performing QC under the spectrum of a centralized model of blood collection and preparation of blood components [32]. This model could provide uniform quality of all blood derivatives and could also relieve local BTSs from a considerable laboratory and economic burden [33]. In Europe, many countries have already implemented centralized QC policies according to the EDQM directives [5,34]. In our country, less than one-third of the BTSs follow these directives, which can be attributed to the decentralized management of preparation of blood components and the lack of the necessary medical technology, especially in the smaller BTSs.

Pharmaceutic hemostatic agents such as fibrinogen concentrate and PCC have been widely incorporated into current therapeutic algorithms for the treatment of massive hemorrhage in trauma patients, intraoperatively, and in cases of obstetric hemorrhage [35,36]. Their use is widespread in many countries and numerous studies have demonstrated their clinical benefits over FFP [37,38,39]. The low rate of use of hemostatic agents recorded in our study may not reflect the true picture, since the provision of these agents may not be carried out exclusively through BTSs. Nevertheless, strengthening the use of pharmaceutic hemostatic agents could improve patients’ management in our country.

## Conclusion

This is the first national study regarding FFP use in Greece. Internal medicine and surgery departments as well as ICUs are the major FFP consumers; thus, their personnel could be the first to be trained in guidelines for proper FFP use. The reasons for FFP requests include not only situations included in the national guidelines but also other situations that, according to these guidelines, are not considered to be indications for FFP transfusion. Various practices regarding plasma production have been noticed; almost every hospital has its own blood establishment and most of the transfused units are produced in-house. FFP is mainly produced from male donors. The main reasons for discarding FFP are not meeting the quality requirements and the returning of thawed plasma that was not finally transfused. Pharmaceutical hemostatic agents are not widely used in all Greek hospitals. Centralization in the production and distribution of blood components could help in ensuring uniformity in all practices and better inventory management.

## Figures and Tables

**Table 1:**
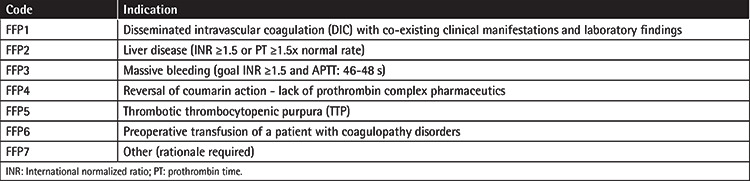
List of coded FFP transfusion indications.

**Table 2:**
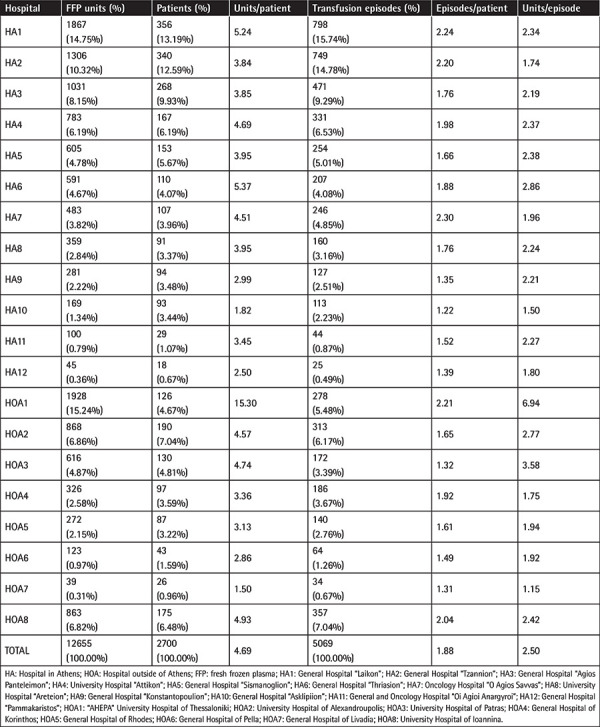
Participating hospitals, respective FFP units, episodes of transfusion, and units/episode in each hospital.

**Table 3:**
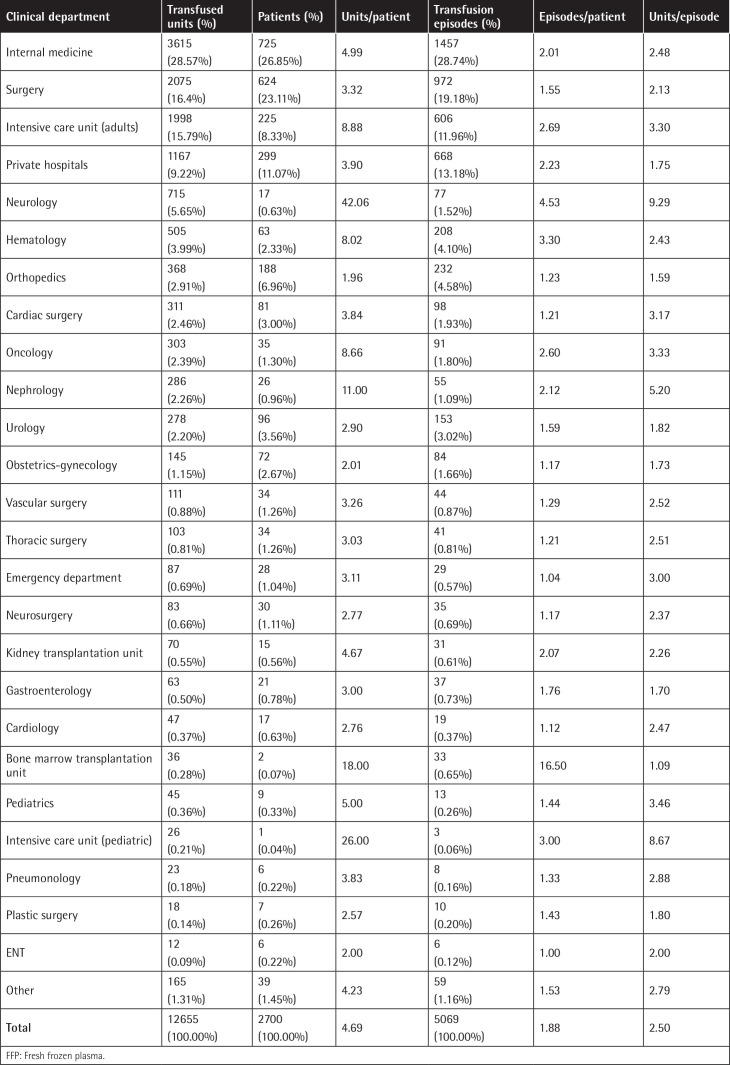
Distribution of patients who received FFP grouped by clinical department.

**Table 4:**
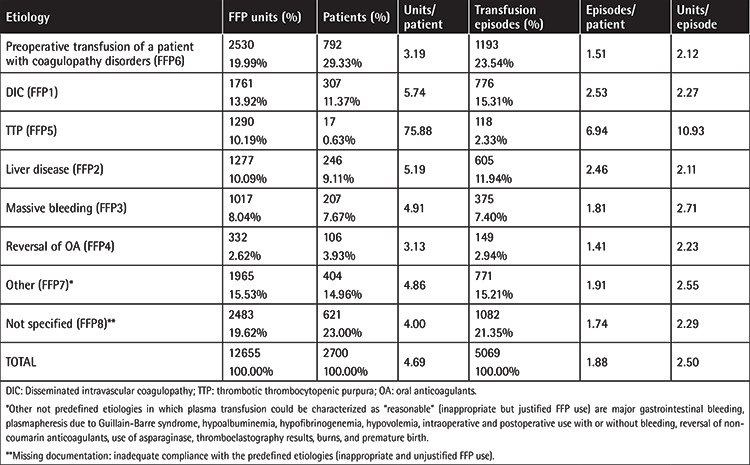
Etiology for FFP transfusions, numbers of patients, transfusion episodes, units/patient, episodes/patient, and units/episode.

**Table 5:**
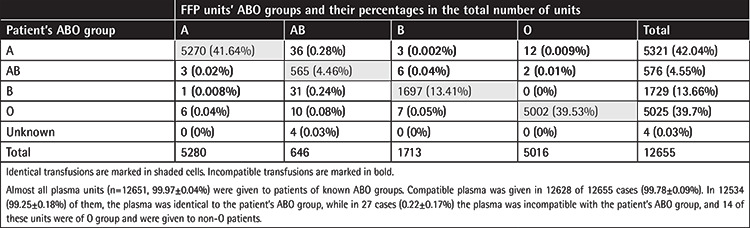
ABO groups of FFP units and how they were used.

**Table 6:**
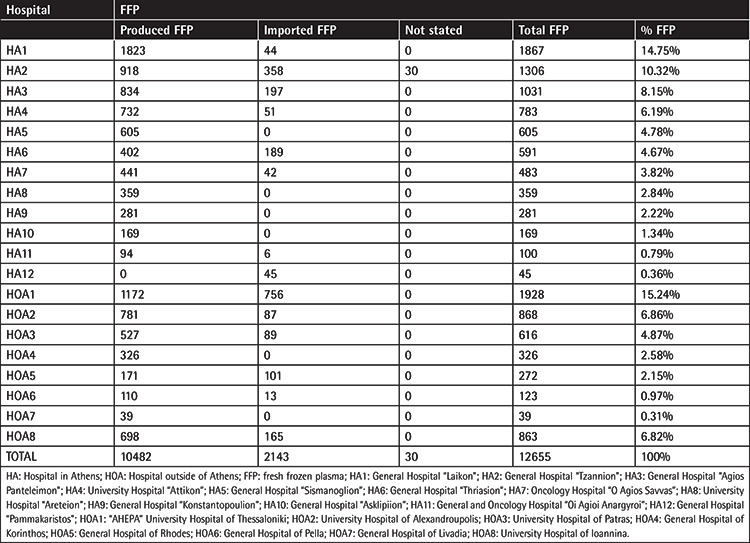
FFP units produced in house vs. imported FFP units.

**Table 7:**
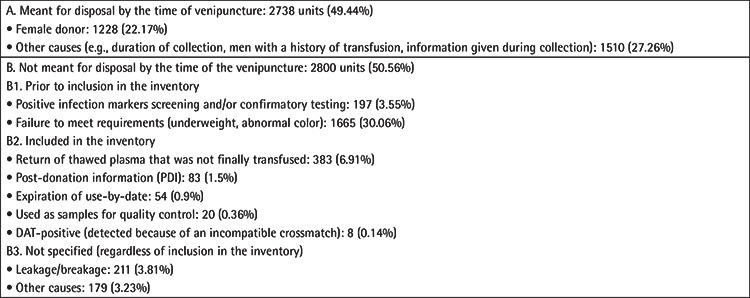
Reasons for plasma to be discarded.
